# Participant Perceptions of a University Continuing Education Intervention Addressing Job Burnout and Self-Care Strategies

**DOI:** 10.3390/ijerph23020263

**Published:** 2026-02-20

**Authors:** Brandon Workman, Laura Nabors, Samuel Adabla

**Affiliations:** 1 Department of Environmental and Public Health Sciences, University of Cincinnati, Cincinnati, OH 45267, USA; 2 Health Promotion & Education Program, School of Human Services, University of Cincinnati, Cincinnati, OH 45221, USA; naborsla@ucmail.uc.edu (L.N.); samuela@hcmhrsb.org (S.A.); 3 Hamilton County Mental Health & Recovery Services Board Cincinnati, OH 45219, USA

**Keywords:** adult learner, job burnout, workplace stress, safety professionals, continuing professional education, mixed methods

## Abstract

**Highlights:**

**Public health relevance—How does this work relate to a public health issue?**

- Burnout has become a prominent psychosocial occupational hazard among today’s workforce that demands urgent attention. Unmanaged job burnout can contribute to heightened personal and task-related conflicts among colleagues, while also increasing the risk of poor mental health outcomes and suicidal ideation for some workers.
- Providing educational resources and well-being interventions that address resilience and strategies for self-care may positively impact employees by improving work engagement and quality of life while reducing stress, anxiety, and depressive symptoms.

**Public health significance—Why is this work of significance to public health?**

- The current study adds to the literature by providing a novel account of a training program that equipped employees from underrepresented industry sectors, i.e., the construction and occupational health and safety sectors, with skills from the evidence base, including learning the signs of job burnout and mental health concerns, as well as interventions to support well-being and self-care while at work.
- Results of the training showed that participants learned new approaches to promote physical, emotional, and psychological well-being in the workplace to improve their self-care behaviors.

**Public health implications—What are the key implications or messages for practitioners, policy makers and/or researchers in public health?**

- Continuing professional education initiatives aimed at managing job-related stress can assist employees with recognizing and managing burnout and enhance both productivity and overall well-being.
- Results of the needs assessment presented in the current study hold promise for guiding the design and implementation of future continuing professional education initiatives.

**Abstract:**

**Objective**: The current study assessed outcomes of a continuing professional education program aimed at managing job-related stress to assist employees with recognizing and managing burnout and enhancing both productivity and overall well-being. **Study Design**: This study outlines the implementation of a needs assessment survey and the development of a non-credit training course for working professionals that addressed risks of burnout, suicidality, and self-care strategies to support mental health in the workplace. **Methods**: The sample for the current study consisted of 398 predominantly mid- to senior-level professionals. Participants were divided into two cohorts. The first cohort completed a structured needs assessment survey between June 2023 and July 2023 and provided ideas for curriculum development. The second cohort participated in synchronous, instructor-led virtual training sessions and completed pre- and post-training questionnaires between January 2024 and June 2024. A mixed-method content analysis was conducted to identify recurring themes and their frequency in course questionnaires. **Results**: Findings suggest that the training successfully expanded participants’ understanding of signs of burnout and of new approaches to improve well-being in the workplace including forming friendships, engaging in mindfulness activities, and taking time off for a mental health day. **Conclusions**: Future research should explore the long-term impacts of such interventions and compare delivery methods, including virtual and in-person formats, to determine the most effective approaches for promoting mental well-being at work.

## 1. Introduction

Declining mental health among the workforce is one of the main reasons for high rates of absenteeism (i.e., being absent from work), presenteeism (i.e., diminished productivity while at work), and burnout [1,2,3]. Burnout is a work-related psychological condition, often characterized by emotional exhaustion, feelings of cynicism or detachment, and a low sense of personal accomplishment [4,5]. Burnout has become a prominent psychosocial occupational hazard among today’s workforce that demands urgent attention [1]. Unmanaged job burnout can contribute to heightened personal and task-related conflicts among colleagues, while also increasing the risk of poor mental health outcomes and suicidal ideation for some workers [6,7,8]. Further, burnout may lead to increased employee turnover, creating skill gaps and high costs within organizations [9,10]. Workplace resources, policies, and training interventions, aimed at managing job-related stress can assist employees with recognizing and managing burnout and enhance both productivity and overall well-being [11,12].

Providing resources and well-being interventions that address resilience and strategies for self-care may positively impact employees by improving work engagement and quality of life while reducing stress, anxiety, and depressive symptoms [13,14]. Job resources encompass improved awareness education, Employee Assistance Programs (EAP), and enhanced options for supervisor support, co-worker support, and job control [13,15,16]. Personal resources can incorporate education for employees focusing on self-care strategies, which encompass behaviors and practices that support physical, emotional, and psychological well-being [17,18]. Self-care strategies may help reduce burnout and stress while improving job performance and job satisfaction [13,19]. Notably, burnout is primarily driven by work-related factors and, as a result, cannot always be resolved through self-care practices [20]. Nonetheless, self-care addresses a wide range of activities people perform such as healthy nutrition, exercise, mindfulness, maintaining a good sleep schedule, engaging in hobbies or leisure activities, and seeking social support to promote overall health and well-being [17,18,21].

A comprehensive, multifaceted approach is essential to effectively reduce burnout, minimize turnover, and promote overall well-being for employees [12]. It is imperative to implement and evaluate tailored interventions that address the unique mental health challenges faced by the workforce [22]. Health care professionals were disproportionately represented in burnout-related research, especially during the COVID-19 pandemic [23,24]. However, individuals working across all occupational sectors are susceptible to the effects of burnout [5,25,26]. Thus, a critical research gap remains in effectively assessing the mental health needs of underrepresented employees outside of the health care industry in job burnout research. For example, limited research is available exploring the impact of job burnout among firefighters and occupational health and safety specialists [27,28]. Among these professions, burnout has been linked to safety behavior deviations and violations such as personal protective equipment compliance, adherence to safe work practices, and safety reporting and communication [27]. To meet the needs of a diverse workforce, more empirical research is vital for creating tailored training programs that address specific mental health concerns, while considering factors like demographics, job roles, and individual needs [29].

Therefore, the purpose of this study was to present findings from a non-credit continuing professional education course addressing job burnout, mental health challenges, suicidality, and self-care practices. The course was provided for a broad workforce at risk for safety concerns and environmental exposures. This study is guided by two main aims: (1) to outline the development of a worker-informed training program designed to support workplace well-being and (2) to examine participant perceptions before and after the training, which focuses on recognizing and managing job burnout and promoting self-care strategies. The specific objectives were as follows: (a) to describe the findings of a needs assessment that guided the creation of a training course; (b) to evaluate change in perceptions of job burnout and effective strategies for self-care among diverse occupation groups after training; and (c) to inform future training efforts related to job burnout.

## 2. Materials and Methods

### 2.1. Participants

The sample for the current study consisted of 398 participants who had previously completed non-credit training for health and safety through a university continuing education program. Participants included occupational health and safety specialists, industrial hygienists, public health practitioners, and other professionals pursuing continuing education units in topics addressing occupational hazards and environmental exposures. Participants were divided into two cohorts. The first cohort (*n* = 268) completed a structured needs assessment survey between June 2023 and July 2023 and provided ideas for developing the curriculum for virtual training. Next, the second cohort of participants (*n* = 130) completed one of seven virtual training sessions conducted between January 2024 and June 2024. Course participants were surveyed at the time of enrollment through a pre-study survey and again upon completion of the course through a post-study survey. Responses were collected in the form of open and closed-ended questions. Prior to participating in the needs assessment and training course, participants read an informed consent statement that explained the purpose, procedures, and perceived benefits of participation. The study protocol was approved by the University of Cincinnati Institutional Review Board (2024-0563) and determined to be non-human subjects research.

### 2.2. Procedures

#### 2.2.1. Needs Assessment

Participants received an email inviting them to complete a needs assessment survey to identify areas in need of training. The email described the consenting process and provided information about the study. Participants accessed a Survey Monkey^®^ link to submit their responses. The email and survey link were shared with participants via a listserv consisting of members of the workforce who had previously attended non-credit courses at a university program for health and safety continuing education. The survey was anonymous, and no demographic data were collected. Participants were asked to identify training topics that would most benefit their personal and professional development (Table 1).

#### 2.2.2. Course Implementation

Previous research addressing burnout, suicide prevention, and mindfulness interventions targeting employees suggest that training programs are effective when delivered over a few hours [29,30] to eight weeks [31]. As such, a 3-h, synchronous instructor-led course was created by the authors using open-access resources on the promotion of mental health, reducing risks for depression and suicide, reducing risks for burnout and workplace violence, and improving self-care behaviors [4,32,33,34,35,36]. Participants were provided with information for recognizing burnout among coworkers and ideas for self-care activities that could be used in the workplace. The course also highlighted the benefits of social connectedness and offered ideas to improve work–life balance. Additionally, ideas for improving communication in the workplace and with regulatory agencies were discussed in the course. There were seven courses conducted during the study time frame (January and June 2024). Participants learned of the training course through an announcement that was shared electronically to a university listserv that contained the email addresses of professionals who had previously completed non-credit training for health and safety through a university continuing education program. Participants registered to attend one course offering by accessing a secure registration link, where they read the course description, learning objectives, and an informed consent statement. Following registration participants were provided with a Zoom^®^ invitation for the specified dates of training. After accessing the link for the study, participants completed a survey (via Survey Monkey^®^) to assess demographic information and their ideas about worker behavior related to burnout in the workplace. Participants also listed ideas for improving self-care in the workplace. Upon completing the training, participants accessed a second survey addressing ideas about behavior related to burnout and self-care. They also completed questions evaluating the training, including whether the training improved their knowledge of mental health concerns and how to address them to promote resilience in the workplace and reduce mental health concerns, such as depressive and anxiety symptoms, related to suicidality. Upon completion of the course, participants were provided with a guide sent to them by email that contained relevant phone numbers for resources and consultations.

### 2.3. Data Analyses

Descriptive statistics including frequencies and percentages were calculated to provide information for responses on the needs assessment survey and demographic variables provided on the pre- and post-course questionnaires (IBM SPSS software Version 29). A mixed-method content analysis was conducted to identify recurring themes and their frequency in course questionnaires, aiming to illustrate the relative magnitude and patterns of participants’ responses on the pre- and post-surveys. Open-ended responses to the course questionnaires were subjected to thematic analysis, which involved an open coding approach using memos to record ideas and constant comparative analysis of the questionnaires to determine relevant categories that emerged in the data that could be classified as patterns or themes across the data [37]. Analysis was conducted by [author 1], with key results and interpretation discussed with [author 2] during the analysis. The two coders [authors 1 and 2] examined responses and identified and organized the themes and representative quotes into tables. Discrepancies regarding theme development and interpretation were resolved through further checking [author 3], theme refinement [authors 1, 2, and 3], and co-author discussion [38]. The final rounds of data review and coding generated the finalized set of codes and subcodes presented in the current study. Categories, frequencies, and percentages for the themes that emerged before the training were compared with those that emerged after the training.

## 3. Results

The sample for the current study included 398 participants who were divided into two cohorts. The first cohort (*n* = 268) completed a structured needs assessment survey (June 2023 and July 2023) and provided ideas for developing the curriculum for virtual training. Survey responses led to the development of a training course addressing occupational stressors that negatively impact mental health, including dangerous working conditions, stress, job burnout, and suicide awareness. The second cohort (*n* = 130) completed one of seven virtual training sessions offered between January 2024 and June 2024.

### 3.1. Needs Assessment

The 268 participants comprising the first cohort were asked to identify training topics that would most benefit their personal and professional development (Table 1). The assessment guided the creation of a training course, examined in the current study, aimed at supporting workplace well-being by helping participants recognize and manage job burnout and adopt effective self-care strategies. Demographic details were not collected from participants who completed the needs assessment. The types of training identified as most needed include compliance with regulatory standards (*n* = 79, 29%), leadership skills (*n* = 36, 13%), disaster preparedness (*n* = 131, 49%), fall protection (*n* = 177, 66%), working in temperature extremes (*n* = 160, 60%), and other general topics related to health and safety (*n* = 46, 17%). Participants were also interested in receiving information for suicide prevention (*n* = 128, 48%), work–life balance and tips for preventing workplace burnout and fatigue (*n* = 142, 53%), and approaches for avoiding workplace violence (*n* = 139, 52%; see Table 1).

### 3.2. Course Evaluation

Following course development, a second cohort of participants (*n* = 130) registered to attend a course by accessing a secure registration link that was shared electronically to a university listserv. The course was deployed seven times during the study timeframe (January and June 2024). After accessing the link for the study, participants completed a pre-training survey (via Survey Monkey^®^) to assess demographic information and their ideas about worker behavior related to burnout in the workplace. Participants also listed ideas for improving self-care in the workplace. Upon completing the training, participants accessed a second survey addressing ideas about behavior related to burnout and self-care. The demographic characteristics of participants who completed the training course are described in Table 2. Most participants were White (66.2%), male (53.1%) and were 41 years of age or older (77.7%). Small and large companies were equally represented in the sample with 44.6% of participants working at a company with 1 to 100 employees and 47.7% of participants working at a company with 101 to 250+ employees (see Table 2). Participants fell into the following job classifications, executive (19.2%), supervisor (36.2%), first-level manager (11.5%), experienced staff (29.2%), or entry level staff (3.8%). Most participants (80%) did not have a trained mental health professional on staff or did not know if they had a mental health professional on staff. Participants did not have dedicated meeting free days, and over half of participants were not offered a mental health day to alleviate stress. Most participants were encouraged to take breaks at their company to reduce stress (Table 2).

### 3.3. Results of the Pre-Training Survey

#### 3.3.1. Perceptions of Workplace Burnout Pre-Training

Prior to the training, participants provided their ideas for negative behaviors a coworker might exhibit that would be associated with workplace burnout (Table 3). All participants (*N* = 130) endorsed changes in work style as an indicator of burnout. They believed that lower work productivity and performing work poorly (*n* = 39, 30%), carelessness (*n* = 29, 22%), being late or absent (*n* = 29, 22%), and having a negative outlook on work and coworkers (*n* = 33, 26%) would indicate that a coworker was experiencing workplace burnout (see Table 3). Regarding carelessness, Participant #20 (P20) described a coworker experiencing burnout as having, “lack of self-care, lack of care for others’ needs, lack of punctuality and/or attendance, decreased interaction with co-workers, loss of pride in work products, insomnia, tardiness, inability to focus, and personality changes.” Participants mentioned physical changes, such as exhaustion (*n* = 32, 25%) as another indicator of workplace burnout. Additionally, changes in mental health (*n* = 110, 85%) were identified by participants. Participants described a coworker who was experiencing workplace burnout as someone who was angry (*n* = 28, 22%), had a bad attitude (*n* = 21, 16%), anxiety (*n* = 19, 15%), frustration (*n* = 5, 4%), feelings of hopelessness (*n* = 13, 10%), discussed suicide (*n* = 6, 5%), and had difficulty focusing (*n* = 18, 13%; Table 3). P39 noted, “emotional exhaustion, irritability, isolation, depression, missing work, not performing well on normal job duties, feeling of being overloaded with work, on the breach of being fired,” as signs of burnout. Additionally, some participants identified acting out behavior as a sign of burnout (*n* = 33, 25%). The following subthemes were endorsed, bullying (*n* = 5, 4%), negative outbursts to minor issues (*n* = 11, 8%), physical altercations (*n* = 4, 3%), and verbal altercations (*n* = 13, 10%; Table 3).

#### 3.3.2. Participants’ Ideas for Self-Care on the Job Pre-Training

Before completing the training, participants provided ideas for self-care in the workplace (Table 4). Participants supported maintaining a good work environment (*n* = 62, 48%) to support self-care by taking allotted breaks (*n* = 48, 37%) and using paid time off (PTO; *n* =14, 11%). P98 suggested “taking breaks and lunches as scheduled,” as important for self-care on the job. Participants provided ideas for a positive work–life, including maintaining a work–life balance and setting achievable goals (*n* =18, 14%). P73 suggested creating a ‘shutdown’ routine at the end of the work to separate work from personal life obligations. Several suggested having a positive mindset (*n* = 7, 5%) and using mental health resources at the workplace, including utilizing the EAP and bringing concerns to human resources (HR; *n* = 10, 8%). Participants endorsed having a good line of communication with their managers (*n* = 7, 6%) and asking for help with workload issues (*n* = 8, 6%) as supportive of self-care. P110 mentioned that it was important to, “make sure you let someone know if the workload is too much, or if you do not understand what is being asked of you.” Being health conscious (*n* = 14, 11%) was viewed as important for self-care. Participants suggested having a healthy snack and staying hydrated. Participants considered relaxation (*n* = 28, 22%) as an asset to guarding their self-care at work. They provided the following examples, meditation (*n* = 11, 9%), going for a walk (*n* = 13, 10%), and listening to music (*n* = 4, 3%; see Table 4).

### 3.4. Results of the Post-Training Survey

#### 3.4.1. Perceptions of Workplace Burnout Post-Training

Table 5 presents participants’ perceptions of negative behaviors associated with burnout post-training. Some participants identified discrimination and bullying as a sign of workplace burnout (*n* = 31, 24%; Table 5). This included being critical of coworkers and clients (*n* = 15, 12%) and hostile in the workplace (*n* = 16, 12%). P100 described a coworker who was experiencing workplace burnout as “being cynical or critical at work, becoming irritable or impatient with co-workers, customers, or clients,” and “calling a person out in front of the crew.” Changes in workstyle (*n* = 68, 52%) were endorsed by participants post-training as a contributing factor to workplace burnout. Many of the subthemes echoed the identified themes during the pre-training assessment, such as limited paid time off (*n* = 5, 4%), absenteeism and high turnover (*n* = 14, 11%), lack of productivity (*n* = 16, 12%), reporting to work late (*n* = 8, 6%), and having a bad attitude at work (*n* = 13, 10%). Following the training, a new subtheme for changes in workstyle was identified, increased complacency and possible injury (*n* = 12, 9%). P34 mentioned, “loss of interest, loss of attention, distraction leading to accidents… feeling disillusioned about your job or having a lack of satisfaction in achievements,” as examples of perceptions and ramifications of workplace burnout.

Further, personal and professional relationship problems were viewed as being associated with burnout (*n* = 18, 14%). Subthemes included lack of meaningful relationships with friends and family/isolation (*n* = 8, 6%), domestic problems (*n* = 4, 3%), and social withdrawal (*n* = 6, 5%; Table 5). Specifically, participants mentioned “feeling lonely or isolated” (P26) and “difficulty with relationships outside of work” (P17) as indicators of a colleague experiencing workplace burnout. More types of physical health changes associated with burnout were discussed in more detail post-training, compared to pretraining. Participants mentioned that having a headache, stomachache, or chronic fatigue/illness (*n* = 8, 6%), elevated blood pressure (*n* = 2, 2%), and lack of sleep or sleep interruptions (*n* = 21, 16%) could be signs of workplace burnout. Changes in mental status or mental health (*n* = 90, 70%) were also discussed in more detail, with subthemes including depression (*n* = 22, 17%), anxiety (*n* = 17, 13%), lack of concentration (*n* = 2, 2%), anger (*n* = 15, 12%), lack of motivation (*n* = 4, 3%), feeling stressed (*n* = 9, 7%), discussing suicide (*n* = 7, 5%), and irritability (*n* = 14, 11%). P79 described an employee with “lack of interest, lack of motivation, poor performance, missing work,” as someone who may be experiencing burnout. Participants also identified behavioral changes (*n* = 21, 16%) as signs of a colleague experiencing workplace burnout. Subthemes included drinking alcohol in excess (*n* = 11, 8%), self-medicating (*n* = 6, 5%), and irresponsibility for finances (*n* = 4, 3%; see Table 5).

#### 3.4.2. Participants’ Ideas for Self-Care on the Job Post-Training

Following the training, participants listed new strategies for self-care on the job. For example, participants recognized the importance of forming friendships at work to support their self-care (Table 6). Specifically, communicating with peers and talking with a friend was endorsed by participants (*n* = 17, 13%). P30 mentioned that “talking with co-workers about what is going on” can help improve a difficult situation at work. Participants also learned new relaxation techniques (*n* = 22, 17%) for the workplace including meditation (*n* = 13, 10%), and yoga or breathing exercises (*n* = 9, 7%). P100 highlighted the value of mindfulness, stating “practice mindfulness and being aware of your surroundings without judgment. Meditation can help you practice mindfulness.” A new theme identified post-training was setting boundaries at work (*n* = 36, 27%; Table 6). Related subthemes included keeping work and personal life separate by setting realistic goals (*n* = 24, 18%) and finding employment at companies that respect their employees (*n* = 12, 9%). P122 advised, “set realistic goals for deadlines and expectations of what is achievable date to date.” Some participants (*n* = 49, 37%) also provided more examples of taking breaks to support their self-care. They supported utilizing PTO for vacations (*n* = 11, 8%) and taking a day off for their mental health as needed (*n* = 38, 29%). Furthermore, some participants suggested new ideas for improving employees’ physical and mental health conditions at work. Ideas included getting a good night’s sleep (*n* = 8, 6%), drinking enough water and eating balanced meals (*n* = 9, 7%), and incorporating exercise into their fitness routine (*n* = 17, 13%) in addition to walking. Importantly, participants emphasized the value of seeking professional mental health support, both internal and external to their company (*n* = 18, 14%). Specifically, participants mentioned counseling (*n* = 9, 7%) in addition to utilizing Employee Assistance Programs (*n* = 9, 7%) as ways to achieve this (see Table 6). Certain themes related to self-care strategies appeared in both the pre- and post-training surveys, such as relaxation (*n* = 28, 22% pre-training vs. *n* = 22, 17% post-training), taking breaks to maintain a good work environment (*n* = 62, 48% vs. *n* = 49, 37%), work–life balance (*n* = 18, 14% vs. *n* = 24, 18%), and speaking with the EAP program (*n* =10, 8% vs. *n* = 9, 7%). Although some of these areas were endorsed at lower frequency in the follow-up survey, participants were listing a new broader range of strategies post-training.

## 4. Discussion

The current study used a mixed methods approach to highlight participants’ perceptions of negative behaviors associated with workplace burnout and related mental health problems. Results showed that participants learned new approaches to promote physical, emotional, and psychological well-being in the workplace to improve their self-care behaviors [17]. Following the training, participants recognized the importance of forming friendships at work, practicing mindfulness, and discussed new ideas for setting boundaries to balance professional and personal commitments and to support their self-care. Previous research examining the association between burnout and poor mental health outcomes (exhaustion, suicidal ideation, etc.) has typically focused on health care workers, medical students, and employees in other human services professions [19,39,40,41]. The current study adds to the literature by providing a novel account of a training program that equipped employees from other industry sectors, i.e., the construction and occupational health and safety sectors, with skills from the evidence base, including learning the signs of job burnout and mental health concerns, as well as interventions to support well-being and self-care while at work [42,43,44]. The findings of this study offer valuable insights for guiding the design and implementation of future continuing professional education initiatives. Specifically, the results suggest that interventions that improve work–life balance and add self-care opportunities in the workplace may be valued by employees.

Results of the needs assessment presented in the current study hold promise for guiding the design and implementation of future continuing professional education initiatives. The assessment identified overall training needs and preferences for participants representing a variety of professions. Valuable input was provided by professionals from the construction, occupational health and safety, and education and training sectors. The types of training identified as most needed fall within several broad topic areas, including compliance and legal issues, general topics in health and safety training, and mental health related topics. The topics with the most interest included fall protection, preventing heat-related illness, burnout, workplace violence, disaster preparedness and emergency response, suicide prevention, and compliance with regulatory standards. The outcomes of the needs assessment provide useful direction for informing the selection of relevant and impactful topics of interest for future continuing education programming. However, the assessment excluded demographic data, as well as many other potentially relevant training topics for employees that should be considered in future surveys.

Results indicated that the training provided employees with new knowledge of behavioral changes, such as exhibiting negative attitudes, being complacent on the job, and bullying others as being related to burnout [45,46,47]. Some themes appear in both pre- and post-training surveys recognizing burnout among coworkers (i.e., change in work style and change in mental status or mental health), however, the frequencies were lower at follow-up than initial survey. A lower count at follow-up may indicate a shift in participants’ focus or a deeper internalization of the new concepts covered in the course, or it could be that they were interested in presenting new strategies they learned from the training. After the training participants used new terms to discuss negative coworker behaviors and seemed to differentiate their responses into more categories indicating changed understanding of interpersonal or relationship behavior compared to pre-training. New themes related to relationship problems emerged, including workplace hostility and discrimination. What may be more common characteristics associated with burnout were identified at high levels pre- and post-training. Some of these characteristics were absenteeism and high turnover, lack of productivity, reporting to work late, and having a bad attitude at work. After the training participants provided more detailed comments about physical symptoms such as sleep disturbances and chronic fatigue. Their description of negative behaviors became more refined. For example, at pre-training physical and verbal altercations were mentioned, and at post-test answers were more specific as participants discussed being cynical and critical of coworkers and being discriminatory, angry, or hostile in the workplace as being related to burnout [48]. Participants also recognized more mental health concerns after the training related to burnout, including depression, lack of motivation at work, and irritability [49,50]. Additionally, participants identified a lack of meaningful relationships with friends and family, along with feelings of loneliness and isolation, as signs of burnout post-training [51,52].

The course helped participants learn new ideas and approaches to support their self-care while at work. As with the themes identified for recognizing burnout, certain themes related to self-care strategies, such as relaxation and the use of workplace mental health resources, appeared in both the pre- and post-training surveys. However, their lower frequency in the follow-up survey suggests that participants learned new concepts from the training. For instance, participants primarily associated self-care with maintaining a good work environment and relaxation prior to the training. Following the training, participants expanded their perspectives on self-care, highlighting a broader range of strategies. These included building social connections in the workplace, establishing professional boundaries, taking breaks or time off, and utilizing available organizational resources. Additionally, participants emphasized improvements in sleep quality, integrating regular physical activity into their routines, and adopting mind–body practices such as yoga to manage stress [14,53,54]. Post-training surveys revealed that a greater number of participants mentioned talking to a counselor as a self-care strategy. Encouraging workers to utilize counseling services, as part of mental health training for employees, may yield significant results for their psycho-social well-being and reduce stigma surrounding the perceptions of mental disorders and receiving counseling services [49,55]. Overall, the greater number of comments related to recognizing burnout and suggestions for self-care following the training may reflect a heightened awareness of the importance of work–life balance, as well as an understanding of the positive effects of mutual respect between employers and employees in the workplace.

### Limitations

This study has several limitations. Demographic data and many other potential topics of training that would benefit the workforce were not included in the needs assessment, mainly due to the length of the assessment. Participants were drawn from diverse occupational fields, rather than a single, centralized profession. Given this heterogeneity, the findings cannot be generalized to any specific occupational group or demographic. Eliciting responses from a larger and diverse population may have identified other topics pertinent to their industries and professions. There was also a possibility of selection bias, as participants were not randomly selected. Longitudinal studies that have a wait list group or an active control group would strengthen the study design and determine the impact of the training. In future studies, researchers may wish to explore the causes and impact of job burnout among a specific underrepresented occupation group, e.g., safety professionals [28]. The current study did not assess current strategies that employers are implementing in workplaces to provide support for mental health problems. Thematic analysis could be subject to researcher bias and participant responses regarding burnout and self-care could be subject to social desirability bias. Additionally, the study is not explicitly guided by an established theoretical framework, limiting its theoretical contribution. Future studies could be strengthened by integrating relevant theoretical perspectives to better inform study design, variable selection, and interpretation of findings. The training was offered synchronously (live), and this may have prevented busy adults with scheduling conflicts from participating. Offering additional asynchronous (pre-recorded) training channels may allow for improved attendance of workers without requiring them to be absent from work [56]. Lastly, the current study adopts a descriptive research approach; it does not allow for causal or explanatory conclusions regarding how increased burnout may lead to specific mental or emotional outcomes. Future research is therefore encouraged to employ explanatory or analytical designs, potentially incorporating thematic analysis or other inferential methods.

## 5. Conclusions

The current study describes the results of a needs assessment and a pre- and post-training survey. The needs assessment identified several broad topic areas that guided our intervention and may be useful for development of future training experiences. Findings also suggest that the training course effectively helped participants recognize signs of burnout and learn additional approaches for supporting self-care. Following the training, participants recognized the importance of forming friendships, engaging in mindfulness activities, and taking time off for a mental health day to support their mental well-being. They also learned new approaches to promote physical, emotional, and psychological well-being at work and approaches to improve self-care practices in the workplace. Importantly, after the training participants supported speaking to a professional, both internal and external to their company for mental health support. Specifically, participants mentioned speaking to a professional counselor for mental health support in addition to utilizing Employee Assistance Programs. Improving the recognition of coworker behaviors may lead to more referrals for care for workers experiencing burnout and stress, and reduce turnover related to a negative environment in the workplace, and these trends are areas for future study. As such, future program planners responsible for developing workforce training interventions should continue to explore the challenges, advantages, and overall effectiveness of delivering virtual and in-person mental health training interventions at worksites. Moreover, research is needed to better understand the interconnections between relational support and burnout in the workplace.

## Figures and Tables

**Table 1:** Topics of General Interest for New Continuing Education Courses Based on Needs Assessment Responses.

Topics	Percent Interest
**Compliance and Legal Issues**	
Compliance with regulatory standards	29%
Leadership and management skills for safety and health professionals	13%
**General Occupational Health Topics of Interest**	
Disaster preparedness and emergency response	49%
Fall protection	66%
Working in temperature extremes	60%
Other, occupational safety	17%
**Mental Health**	
Suicide Prevention	48%
Job burnout/fatigue	53%
Workplace violence	52%

Note: Total needs assessment survey respondents included *N* = 268.

**Table 2:** Demographic Details for Participants Completing the Training Course.

Characteristics (*N* = 130)	*n* (%)
**Race/Ethnicity**	
White	86 (66.2)
Black	22 (16.9)
Asian	2 (1.5)
American Indian/Alaskan Native	3 (2.3)
Hispanic	14 (10.8)
Other, multiracial	3 (2.3)
**Sex**	
Male	69 (53.1)
Female	60 (46.2)
Missing Data	1 (0.8)
**Age**	
18–24	2 (1.5)
25–31	9 (6.9)
32–40	18 (13.8)
41–50	50 (38.5)
51–60	33 (25.4)
61–70	13 (10)
71+	5 (3.8)
**Trained Mental Health Professional on Staff**	
Yes	26 (20)
No	89 (68.5)
Do not know	15 (11.5)
**Meeting Free Days**	
Yes	53 (40.8)
No	55 (42.3)
Do not know	22 (16.9)
**Breaks Encouraged**	
Yes	102 (78.5)
No	15 (11.5)
NA	13 (10)
**Offered Mental Health Days Off**	
Yes	41 (31.5)
No	70 (53.8)
Do not know	19 (14.6)
**Job Classification**	
Executive or senior management	25 (19.2)
Supervisor/middle management	47 (36.2)
First-level management	15 (11.5)
Intermediate or experienced staff	38 (29.2)
Entry-level staff	5 (3.8)
**Company Size** (# of employees)	
Under 25	23 (17.7)
26 to 50	17 (13.1)
51 to 100	18 (13.8)
101 to 200	24 (18.5)
201 to 249	11 (8.5)
Over 250	27 (20.8)
NA	10 (7.7)
**Occupation Group**	
Community and social services	4 (3.1)
Construction trades	41 (31.5)
Education and training	13 (10)
Emergency services	2 (1.5)
Government	6 (4.6)
Health care	6 (4.6)
Insurance claims processing	5 (3.8)
Manufacturing	9 (6.9)
Occupational health and safety	23 (17.7)
Oil & gas	4 (3.1)
Telecommunications	4 (3.1)
Other, not applicable	13 (10)

**Table 3:** Participants’ Perceptions of Negative Behaviors Associated with Workplace Burnout Pre-Training.

Theme (Count, %)	Subtheme (Count, %)	Representative Quotes
**Change in workstyle** **(** ***N* = 130, 100%)**		
	Lower work productivity or output (*n* = 39, 30%)	“Irritability, low productivity, increased stress, performing work poorly.” (P57)
		“… routine (going through the motions), negative efficiency output (quality, speed, safety).” (P106)
	Careless (*n* = 29, 22%)	“Lack of self-care, lack of care for others’ needs, lack of punctuality and/or attendance, decreased interaction with co-workers, loss of pride in work products, insomnia, tardiness, inability to focus, and personality changes.” (P20)
		“Employee seems distracted, tired, loses focus; becomes more of a safety hazard to themselves and others, no longer caring as much, makes odd remarks of potential self-harm, new problems may appear in personal lives, marriage or relationship issues, family issues, etc.” (P26)
	Late or missing work (*n* = 29, 22%)	“Calling off or coming in late, having a bad attitude, not having good work performance.” (P110)
	Negative outlook on work and coworkers (*n* = 33, 26%)	“… creating additional stress in the workplace, poor job performance.” (P2)
		“Poor attitude in the workplace and about others in the workplace, the organization, withdrawn.” (P89)
**Physical change** **(** ***n* = 32, 25%)**		
	Exhaustion (*n* = 32, 25%)	“Fatigue, absenteeism, low productivity, aggression, low engagement.” (P88)
		“Exhaustion, depression, no time for non-work-related things, dreading work, trouble sleeping, short temper.” (P73)
**Change in mental status or mental health (** ***n* = 110, 85%)**		
	Anger (*n* = 28, 22%)	“Passive aggressive, aggressive behavior, depressing, hard to work with, does not invest in projects, quick to leave and last to arrive.” (P112)
	Bad attitude (*n* = 21, 16%)	“Disinterest, sleeping, frequent days missed, poor attitude towards company/coworkers, change in eating habits, use of drugs/alcohol.” (P113)
		“Unwillingness to listen to ideas, little to no interaction with co-workers beyond what is required.” (P12)
	Anxiety (*n* = 19, 15%)	“Irritability, low productivity, increased stress, performing work poorly.” (P57)
	Frustration (*n* = 5, 4%)	“Disengagement, anger, anxiety, work stoppage or slowing production, sabotage, quitting.” (P32)
	Feelings of hopelessness (*n* = 13, 10%)	“Fatigue, emotional exhaustion, irritability, isolation, depression, missing work, not performing well on normal job duties, feeling of being overloaded with work, on the breach of being fired.” (P39)
	Discussing suicide, suicidality (*n* = 6, 5%)	“Apathy, suicidal tendencies, tiredness, unnecessary risk-taking, tardiness/absences, argumentative/disgruntled, substance-abuse.” (P105)
	Trouble focusing (*n* = 18, 13%)	“Lack of focus, creativity, and engagement, hostility, bitterness, disagreeableness.” (P103)
**Acting out behavior (** ***n* = 33, 25%)**		
	Bullying (*n* = 5, 4%)	“… violence, bullying, increased liability.” (P10)
	Negative outbursts to minor issues (*n* = 11, 8%)	“Irrational vocal outbursts or behavior acted out.” (P16)
	Altercations, physical (*n* = 4, 3%)	“Lashing out, destructive.” (P15)
		“Low production, disgruntled, low self-esteem, temperament.” (P7)
	Altercations, verbal (*n* = 13, 10%)	“Negative talk, venting.” (P66)
		“… arguments with co-workers or managers.” (P47)

Note. Total survey respondents included *N* = 130.

**Table 4:** Participants’ Ideas for Self-Care on the Job Pre-Training.

Theme (Count, %)	Subtheme (Count, %)	Representative Quotes
**Maintain a good work environment (** ***n* = 62, 48%)**		
	Take allotted breaks (*n* = 48, 37%)	“Taking breaks and lunches as scheduled.” (P106)
		“Stretching, breathing, and walking breaks.” (P102)
	Use time off (*n* = 14, 11%)	“Use personal time off (PTO) and take time off.” (P98)
		“Use vacation time regularly.” (P107)
**Improving work life (** ***n* = 18, 14%)**		
	Maintain work–life balance and setting achievable goals (*n* = 18, 14%)	“Only dealing with work during work hours.” (P79)
		“Create a shutdown routine at the end of the workday.” (P73)
**Self-compassion (** ***n* = 7, 5%)**		
	Positivity (*n* = 7, 5%)	“Focus on the positive and not the negative.” (P106)
**Use mental health resources at the workplace (** ***n* = 10, 8%)**		
	Utilize the Employee Assistance Program (EAP) and bring concerns to human resources (*n* = 10, 8%)	“Feels safe taking advantage of Employee Assistance Program (EAP) services and resources provided by employer.” (P26)
		“Bringing your concerns to human resources.” (P36)
**Communication at work (** ***n* = 15, 12%)**		
	Talk to your manager (*n* = 7, 6%)	“… open communication and discuss issues with supervisor.” (P1)
	Discuss workload issues and ask for help if you do not understand a task (*n* = 8, 6%)	“Make sure you let someone know if the workload is too much, or if you do not understand what is being asked of you.” (P110)
**Be health conscious (** ***n* = 14, 11%)**		
	Have a healthy snack and stay hydrated (*n* = 14, 11%)	“Healthy snacking.” (P115)
		“Drink more water.” (P8)
**Relaxation (** ***n* = 28, 22%)**		
	Meditation (*n* = 11, 9%)	“Breathing exercises and meditation.” (P93)
	Walking (*n* = 13, 10%)	“Taking a walk to reduce stress.” (P61)
		“… walk during a lunch break.” (P16)
	Listen to music (*n* = 4, 3%)	“… music while you’re working.” (P102)

Note. Total survey respondents included *N* = 130.

**Table 5:** Participants’ Perceptions of Negative Behaviors Associated with Workplace Burnout Post-Training.

Theme (Count, %)	Subtheme (Count, %)	Representative Quotes
**Discrimination and bullying (** ***n* = 31, 24%)**		
	Critical of coworkers and clients (*n* = 15, 12%)	“Being cynical or critical at work, becoming irritable or impatient with co-workers, customers, or clients.” (P100)
		“Less empathy while dealing with others.” (P72)
	Workplace hostilities (*n* = 16, 12%)	“Calling a person out in front of the crew.” (P75)
		“Disassociated with groups.” (P116)
**Change in workstyle (** ***n* = 68, 52%)**		
	No-time off (*n* = 5, 4%)	“Extra load of hours of work, no time off.” (P1)
	Absenteeism/turnover (*n* = 14, 11%)	“Not wanting to go to work, increased absenteeism.” (P52)
	Lack of productivity (*n* = 16, 12%)	“Productivity drops, not as innovative or can make more mistakes.” (P59)
	Reporting to work late (*n* = 8, 6%)	“Is late or often misses work.” (P61)
	Bad attitude at work (*n* = 13, 10%)	“Disgruntled, aggressiveness.” (P105)
	Increased complacency and possible injury (*n* = 12, 9%)	“Loss of interest, loss of attention, distraction leading to accidents.” (P33)
		“Feeling disillusioned about your job or having a lack of satisfaction in achievements.” (P100)
**Relationship problems (** ***n* = 18, 14%)**		
	Lack of meaningful relationships with friends and family/isolation (*n* = 8, 6%)	“Feeling lonely or isolated.” (P26)
	Domestic problems (*n* = 4, 3%)	“Difficulty with relationships outside of work.” (P17)
	Withdrawal (*n* = 6, 5%)	“Social withdrawal.” (P83)
**Physical changes (** ***n* = 31, 24%)**		
	Having a headache, stomachaches, or chronic fatigue/illness (*n* = 8, 6%)	“Feeling drained, more likely to get sick.” (P102)
		“Chronic illness.” (P57)
	Elevated blood pressure (*n* = 2, 2%)	“High blood pressure.” (P35)
	Lack of sleep/sleep interruptions (*n* = 21, 16%)	“Sleep issues—too much or too little.” (P40)
**Change in mental status or mental health (** ***n* = 90, 70%)**		
	Depression (*n* = 22, 17%)	“Emotional exhaustion, feelings of depression.” (P122)
	Anxiety (*n* = 17, 13%)	“Anxiety disorders, panic.” (P40)
	Lack of concentration (*n* = 2, 2%)	“Disassociation, lack of concentration.” (P34)
	Anger (*n* = 15, 12%)	“Anger, mood swings.” (P129)
		“Short tempered.” (P37)
	Lack of motivation (*n* = 4, 3%)	“Lack of interest, lack of motivation, poor performance, missing work.” (P79)
	Stressed (*n* = 9, 7%)	“Stress in and outside of work.” (P13)
		“Stress response.” (P56)
	Discussing suicide, suicidality (*n* = 7, 5%)	“Suicide ideations.” (P7)
		“Talk about ways to self-harm.” (P61)
	Irritability (*n* = 14, 11%)	“Irritable, argumentative.” (P14)
		“Becoming distant, irritable, frustrated.” (P26)
**Behavioral changes (** ***n* = 21, 16%)**		
	Drinking alcohol in excess (*n* = 11, 8%)	“Alcohol or substance abuse.” (P92)
	Self-medicating (*n* = 6, 5%)	“Increasing drug use.” (P89)
		“Drug misuse.” (P15)
	Irresponsibility for finances (*n* = 4, 3%)	“Irresponsibility with finances.” (P92)
		“Loss of finances.” (P46)

Note. Total survey respondents included *N* = 130.

**Table 6:** Participants’ Ideas for Self-Care on the Job Post-Training.

Theme (Count, %)	Subtheme (Count, %)	Representative Quotes
**Make friends at work (** ***n* = 17, 13%)**		
	Communicate with peers/talk with a friend (*n* = 17, 13%)	“Talking with co-workers about what is going on.” (P30)
		“Peer-support training for co-workers to become approachable within a judgement-free, confidential, relationship.” (P16)
**Relaxation (** ***n* = 22, 17%)**		
	Meditate (*n* = 13, 10%)	“Practice mindfulness and being aware of your surroundings without judgment. Meditation can help you practice mindfulness.” (P100)
	Yoga or breathing exercises (*n* = 9, 7%)	“Yoga/exercise and deep breathing.” (P15)
**Set boundaries at work (** ***n* = 36, 27%)**		
	Keep work and personal life separate by setting realistic goals (*n* = 24, 18%)	“Maintain work life balance and set healthy boundaries.” (P56)
		“Set realistic goals for deadlines and expectations of what is achievable date to date.” (P122)
	Work with a company that respects employees (*n* = 12, 9%)	“Take work with companies that respect employees, their ideas and mental health.” (P33)
**Take breaks (** ***n* = 49, 37%)**		
	Consider taking a regular day off and a mental health day off (*n* = 38, 29%)	“Take breaks during the day and outside.” (P100)
		“Use your personal time off (PTO) when needed.” (P22)
	Go on a vacation (*n* = 11, 8%)	“Taking vacations at least two times year.” (P125)
**Physical or health change (** ***n* = 34, 26%)**		
	Sleep (*n* = 8, 6%)	“Take care of yourself and get enough sleep.” (P27)
	Drink water and eat a balanced diet (*n* = 9, 7%)	“Drinking enough water and eat healthy food.” (P73)
	Exercise and be active (*n* = 17, 13%)	“Exercise and getting outdoors.” (P36)
**Refer to internal and external professional resources (** ***n* = 18, 14%)**		
	Get counseling (*n* = 9, 7%)	“Utilize company resources for therapy and talk to a mental health professional.” (P29)
	Speak with the Employee Assistance Program (*n* = 9, 7%)	“…take advantage of resources in the workplace for lowering stress.” (P1)
		“Speaking with the EAP.” (P120)

Note. Total survey respondents included *N* = 130.

## Data Availability

Qualitative responses received on the course questionnaires are available from the corresponding author upon reasonable request, in accordance with ethical and institutional guidelines.
